# VCAN Hypomethylation and Expression as Predictive Biomarkers of Drug Sensitivity in Upper Urinary Tract Urothelial Carcinoma

**DOI:** 10.3390/ijms24087486

**Published:** 2023-04-19

**Authors:** Hao-Lun Luo, Yin-Lun Chang, Hui-Ying Liu, Yen-Ting Wu, Ming-Tse Sung, Yu-Li Su, Chun-Chieh Huang, Pei-Chia Wang, Jei-Ming Peng

**Affiliations:** 1 Department of Urology, Kaohsiung Chang Gung Memorial Hospital and Chang Gung University College of Medicine, Kaohsiung 83301, Taiwan; 2 Department of Pathology, Kaohsiung Chang Gung Memorial Hospital and Chang Gung University College of Medicine, Kaohsiung 83301, Taiwan; 3 Department of Hematology and Oncology, Kaohsiung Chang Gung Memorial Hospital and Chang Gung University College of Medicine, Kaohsiung 83301, Taiwan; 4 Department of Radiation Oncology, Kaohsiung Chang Gung Memorial Hospital and Chang Gung University College of Medicine, Kaohsiung 83301, Taiwan; 5 Institute for Translational Research in Biomedicine, Kaohsiung Chang Gung Memorial Hospital, Kaohsiung 83301, Taiwan

**Keywords:** upper tract urothelial carcinoma, hypomethylation, versican, extracellular matrix, DNA methylation, chemoresistance

## Abstract

Versican (VCAN), also known as extracellular matrix proteoglycan 2, has been suggested as a potential biomarker in cancers. Previous research has found that VCAN is highly expressed in bladder cancer. However, its role in predicting outcomes for patients with upper urinary tract urothelial cancer (UTUC) is not well understood. In this study, we collected tissues from 10 patients with UTUC, including 6 with and 4 without lymphovascular invasion (LVI), a pathological feature that plays a significant role in determining metastasis. Results from RNA sequencing revealed that the most differentially expressed genes were involved in extracellular matrix organization. Using the TCGA database for clinical correlation, VCAN was identified as a target for study. A chromosome methylation assay showed that VCAN was hypomethylated in tumors with LVI. In our patient samples, VCAN expression was also found to be high in UTUC tumors with LVI. In vitro analysis showed that knocking down VCAN inhibited cell migration but not proliferation. A heatmap analysis also confirmed a significant correlation between VCAN and migration genes. Additionally, silencing VCAN increased the effectiveness of cisplatin, gemcitabine and epirubicin, thus providing potential opportunities for clinical application.

## 1. Introduction

The standard treatment for upper urinary tract uroepithelial carcinoma (UTUC) is radical nephroureterectomy (RNU), which involves removing the affected kidney and affected ureter [1]. However, this treatment often has a high recurrence rate. Recent studies suggest that neoadjuvant chemotherapy (NAC) and adjuvant chemotherapy (ACH) may improve outcomes for patients with UTUC [2,3]. The recommended chemotherapy regimen for patients with upper urinary tract uroepithelial carcinoma, according to the European Association of Urology (EAU) guidelines, is the same as that used for bladder urothelial carcinoma [4]. Cisplatin-based NAC is considered the standard of care for patients with urothelial carcinoma of the urinary tract and locally advanced UTUC [5]. However, the loss of renal function after RNU therapy can prevent patients from receiving higher doses of chemotherapy [6,7,8]. Immunotherapy is also a postoperative treatment modality, and EMA recently approved nivolumab as a standalone treatment for muscle-invasive UC patients with >1% PD-L1 expression who are at high risk of recurrence post-radical surgery [9]. Identifying new prognostic factors and treatments, as well as understanding the underlying causes of UTUC, could improve the effectiveness of chemotherapy.

Lymphovascular invasion (LVI), or the presence of cancer cells in the lymphatic or blood vessels, is a poor prognostic factor for UTUC and is associated with low survival rates [10,11]. Studies have shown that patients with LVI in regenerative adenocarcinoma have a more aggressive clinical presentation and are more likely to have early biochemical failure after radical regenerative adenomectomy [12]. LVI has also been linked to worse outcomes in other types of cancer, such as esophageal, breast and colorectal cancer [13,14,15]. Studies have also found that LVI in UTUC is associated with advanced tumor stage, higher tumor grade and lymph node metastasis but not with gender or multifocality [16,17,18,19,20]. Additionally, the location of the tumor invasion may also affect the prognosis in cases of LVI in UTUC [21].

Versican (VCAN) is a large extracellular matrix proteoglycan that has been linked to the proliferation and metastasis of tumors [22]. VCAN is found in various sources in cancer, including tumor cells, tumor-associated stroma, and tumor-associated immune cells [22]. High levels of VCAN have been observed in various types of cancer, including bladder cancer and melanoma [23,24]. Studies have also shown that VCAN promotes the growth and progression of ovarian cancer by increasing the expression of TGFβ by fibroblasts known as cancer-associated fibroblasts (CAF) [25]. Elevated levels of VCAN have been associated with a poorer prognosis in cancer [26]. Furthermore, high levels of VCAN and hyaluronic acid have been linked to a poorer prognosis in ovarian cancer patients. VCAN has also been found to activate inflammatory cells by binding to TLR2 and adhesion molecules, thus leading to the expression of inflammation-regulating cytokines such as TNFα, IL-1β and IL-6. This dysregulation involves specific signaling pathways such as JAK/STAT and PI3-kinase/AKT, with activation of AKT leading to increased β-catenin signaling [26]. VCAN expression has also been identified as a diagnostic and therapeutic target in colon cancer and as a potential biomarker of the tumor microenvironment [27,28].

In our study of ten patients with UTUC, six had pathological LVI while the other four did not. RNA sequencing analysis revealed that tumors with LVI had an increased extracellular matrix composition. Data from the Cancer Genome Atlas revealed a prognostic role for VCAN in carcinogenesis. Further analysis of tumor tissue from patients at Kaohsiung Chang Gung Memorial Hospital showed that patients with LVI had significantly higher levels of VCAN. In vitro experiments found that silencing VCAN inhibited cell migration but not cell proliferation. Additionally, silencing VCAN increased the effectiveness of the chemotherapy drugs cisplatin, gemcitabine and epirubicin in urothelial cells. These findings suggest that VCAN plays a role in LVI and that silencing VCAN may improve the efficacy of chemotherapy in UTUC patients.

## 2. Results

### 2.1. RNAseq Analysis Identified VCAN as an Oncogene in LVI-Positive UTUC

**Figure 1:**
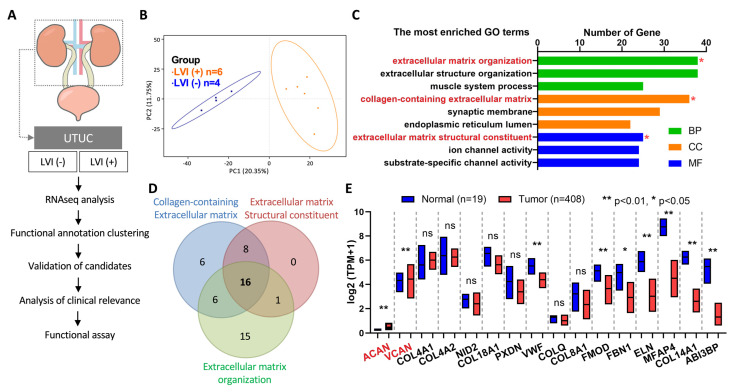
VCAN as a potential oncogene in lymphovascular invasion of UTUC through RNAseq analysis. (**A**,**B**) The flowchart illustrates the process of comparing the RNA profiles of normal and UTUC tumors, specifically examining the RNA expression in UTUC tumor tissues from patients with or without lymphovascular invasion. (**C**,**D**) The results of the RNA analysis were further evaluated using Gene Ontology (GO) analysis to classify the differentially expressed genes based on their molecular function, biological process and cellular component. Red asterisk, functional clustering was performed using these features. (**E**) Statistical analysis of the gene expression levels in normal and bladder cancer samples was conducted to identify genes with significant differences in expression. ns, not significant; * *p* < 0.05; ** *p* < 0.01.

In Taiwan, the underlying causes of LVI in UTUC have not been extensively studied. To gain a better understanding of the factors associated with LVI in UTUC, we collected ten patients with stage III tumors; six were LVI(+) and four were LVI(−). RNA was extracted from the tumor tissue of these patients and analyzed using RNA sequencing to identify any differences in gene expression between the LVI(+) and LVI(−) groups (Figure 1). Based on the results of RNA sequencing, we selected candidate genes that were differentially expressed between LVI(+) and LVI(−) groups. We then analyzed the clinical data of each candidate gene and performed functional assays to further understand their roles in LVI. The experimental design flow is shown in Figure 1A. The principal component analysis (PCA) of the RNA profile revealed that the RNA expression of LVI(+) and LVI(−) samples can be separated into two distinct groups (Figure 1B). To understand the functional classification of these genes, we used the Gene Ontology (GO) database for analysis. The results revealed more significant differences in the biological process (BP) category for genes related to extracellular matrix organization, in the cellular component (CC) category for genes related to collagen-containing extracellular matrix and in the molecular function (MF) category for genes related to extracellular matrix structural constituents (Figure 1C).

To identify potential marker proteins that can distinguish LVI(+) from LVI(−), we further analyzed the 16 candidate genes that were found to be differentially expressed in the functional assay. These genes include aggrecan (ACAN), versican (VCAN) and 14 other genes (Figure 1D). We then used the TCGA bladder cancer (BLCA) database to evaluate the expression of these genes in patients with tumors. The results showed that the expression of ACAN and VCAN was significantly higher in cancer patients than in normal patients (*p* < 0.01) (Figure 1E). To summarize, RNA sequencing analysis identified candidate genes with differential expression between LVI(+) and LVI(−) groups in UTUC. ACAN and VCAN were not only associated with extracellular matrix composition but were also expressed higher in cancer patients. Hence, they are potential marker proteins for further analysis.

### 2.2. Association of VCAN Expression with Progression of BLCA in TCGA Database

**Figure 2:**
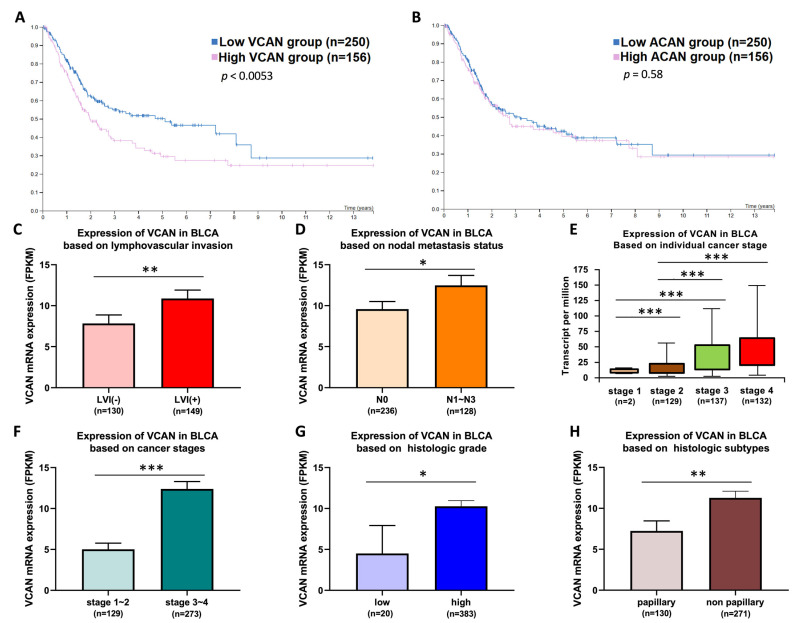
Positive correlation between VCAN expression and poor prognosis, advanced tumor stage, lymph node metastasis and histologic subtypes of UTUC. (**A**,**B**) Survival analysis of patients with high or low expression of VCAN or ACAN in the BLCA database (n = 406) using a log-rank test (log-rank test, *p* < 0.01 and 0.58, respectively). (**C**) High expression of VCAN was significantly associated with lymphovascular invasion in BLCA (*p* < 0.01). (**D**) High expression of VCAN was significantly associated with metastatic lymph nodes (*p* < 0.05). (**E**,**F**) High expression of VCAN was significantly associated with advanced stages of BLCA (all, *p* < 0.001). (**G**) High expression of VCAN was significantly associated with high-grade BLCA (*p* = 0.05). (**H**) High expression of VCAN was significantly associated with non-papillary subtypes of UTUC (*p* < 0.01). All statistics were determined using a two-tailed Student’s *t*-test. * *p* < 0.05; ** *p* < 0.01; *** *p* < 0.001.

After the initial screening process, we identified two candidate genes, VCAN and ACAN, that can discriminate between LVI(+) and LVI(−). To further confirm the correlation between the survival and clinical data of these genes in patients, we used the TCGA database to analyze the survival of patients with high and low expression of VCAN and ACAN. The results showed that the survival of patients with high VCAN expression was significantly lower than that of patients with low VCAN expression (*p* = 0.0053) (Figure 2A). In contrast, there was no significant difference in the survival of patients with high ACAN compared to those with low ACAN (*p* = 0.58) (Figure 2B). Further analysis of the clinical data of VCAN according to the TCGA bladder cancer (BLCA) database showed that VCAN RNA expression was significantly higher in the LVI(+) group than in the LVI(−) group (*p* < 0.01) (Figure 2C). VCAN RNA expression was found to be significantly higher in the N1-3 group of patients with tumor lymph node staging compared to the N0 group (*p* < 0.05), suggesting a higher expression of VCAN in those with metastatic lymph nodes (Figure 2D).

Additionally, VCAN RNA expression was observed to increase progressively with the advancement of cancer stage (Figure 2E). The expression of VCAN was also found to be significantly higher in the stage 3-4 group compared to the stage 1-2 group (*p* < 0.001), implying a correlation between VCAN expression and cancer stage (Figure 2F). The results of a comparison of tumor cell differentiation showed that VCAN RNA expression was significantly higher in the highly differentiated groups compared to the less differentiated groups (*p* = 0.05) (Figure 2G). Finally, the tumor histological classification showed a significant increase in VCAN RNA expression in the non-papillary group compared to the papillary group (*p* < 0.01) (Figure 2H). These clinical data have significance in that VCAN expression increases as the severity of BLCA progresses, suggesting VCAN can not only distinguish LVI(+) and LVI(−) genes but also indicate the degree of tumor malignancy in patients.

### 2.3. VCAN Gene Locus in Chromosome 5: 83.471,618-83,582,303 Was Hypermethylated in UTUC Patients

**Figure 3:**
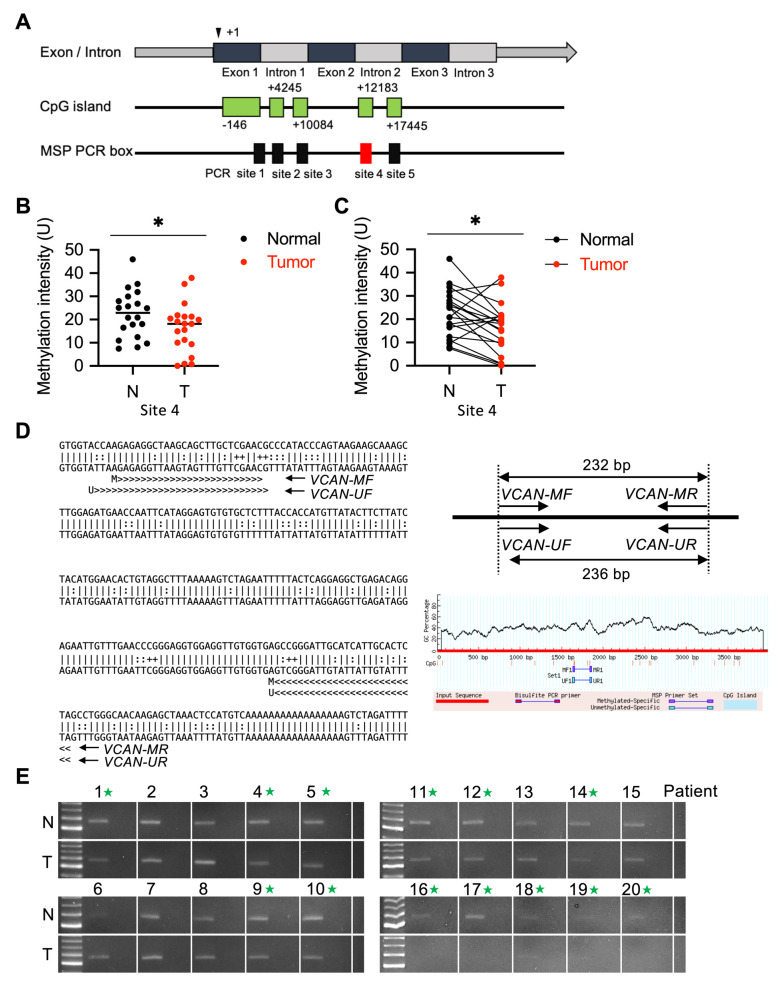
Low methylation of the VCAN in UTUC tumors. The VCAN gene is located on chromosome 5, and CpG islands were identified using the DBCAT and MethPrimer software. Five CpG islands were analyzed using methylation-specific PCR (MS-PCR) to quantify the level of methylation (**A**). The methylation of VCAN in site four was significantly lower in UTUC tumors compared to normal samples (*p* = 0.0145) (**B**). The methylation intensity of N/T pairs in site four also revealed a significant reduction in VCAN (**C**). Schematic of the predicted sequence and primer sets for site four (**D**). DNA agarose gel analysis was performed to quantify the methylation in site four from N/T pairs of UTUC (**E**). Statistical analysis was performed by a paired *t*-test; green asterisk, higher methylation level in the tumor tissues. * *p* < 0.05.

We further investigated the methylation signatures that can detect and distinguish normal tissues from tumors in UTUC. The methylation of VCAN in patients with UTUC tumors was studied using DBCAT and MethPrimer (website: http://dbcat.cgm.ntu.edu.tw and https://www.urogene.org/methprimer/, accessed on 21 March 2022). Five CpG islands were predicted, and methylation-specific PCR (MS-PCR) was performed to determine the methylation level at five sites (Figure 3A). Out of the five predicted methylation sites, site four exhibited a significant difference in methylation levels between normal and tumor tissues. The methylation of VCAN was low in tumors compared to normal samples in site four (Figure 3B) (*p* = 0.0145). The methylation intensity of N/T pairs at site four indicated a significant decrease in VCAN methylation in paired normal and tumor tissues (Figure 3C). The sequence and primer sets were designed by the MethPrimer software (Figure 3D). Through DNA agarose gel electrophoresis analysis, the methylation of VCAN at site four was analyzed and quantified (Figure 3E), with the results of unmethylated VCAN presented in Supplementary Figure S1. The results show that among the 20 patients, 13 had lower methylation of the VCAN gene in their tumor tissues compared to normal tissues.

### 2.4. High Expression of VCAN in LVI-Positive Tumors from UTUC Patients

**Figure 4:**
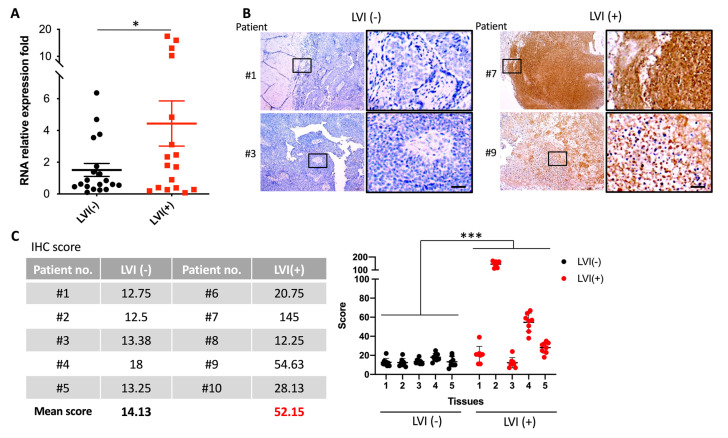
High VCAN expression in LVI-positive tumors from UTUC patients. (**A**) Analysis of VCAN mRNA levels in UTUC patients at the Chang Gung Memorial Hospital revealed that the expression of VCAN mRNA was higher in LVI(+) patients compared to LVI(−) patients. (**B**,**C**) VCAN protein levels in UTUC tumors were quantified through IHC staining using a VCAN antibody, with the examination comprising five LVI(+) and five LVI(−) tumors (*p* < 0.001). Statistical analysis was performed by using two-way ANOVA with Sidak’s multiple comparisons test. * *p* < 0.05; *** *p* < 0.001. Scale bar, 100 µM.

To confirm the utility of the VCAN gene as a marker for distinguishing LVI(+) and LVI(−) in clinical samples, we conducted an experiment that analyzed VCAN RNA expression in 36 stage III UTUC tumors, including 17 LVI(+) and 19 LVI(−) patients. The results revealed that VCAN expression was significantly higher in LVI(+) patients than in LVI(−) patients (*p* = 0.0457) (Figure 4A). Additionally, we evaluated the VCAN protein levels in the tissues of LVI(+) and LVI(−) patients by analyzing VCAN tissue staining of five tumor samples from each group (Figure 4B). Using InForm software analysis, we quantified the intensity of VCAN staining in cancer tissues from five patients in each group and found that the LVI(+) group had a total score of 52.15 compared to 14.13 for the LVI(−) group, indicating that VCAN protein expression was significantly higher in LVI(+) tissues than in LVI(−) tissues (Figure 4C,D). These results suggest that the LVI(+) group exhibited higher RNA and protein expression of VCAN compared to the LVI(−) group in the clinical samples. 

After analyzing clinical data and performing validation in patient tumors, we identified a strong association between VCAN and survival in UTUC as well as the ability to distinguish LVI(+) from LVI(−) through tissue analysis. However, the specific function of VCAN in urothelial carcinoma cells remains unclear. Based on previous research by Said et al., VCAN was found to enhance lung metastasis [23]. Thus, we further investigated the impact of VCAN on cell proliferation and migration in urothelial cancer cells.

### 2.5. VCAN Promoted the Migration of UTUC Cancer Cells

**Figure 5:**
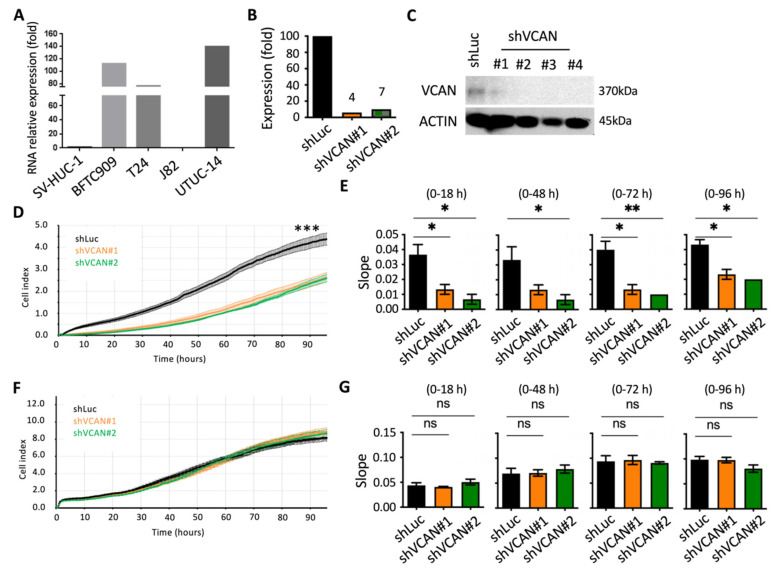
VCAN promoted the migration of cancer cells from UTUC. (**A**) VCAN expression was analyzed in different urothelial cancer cell lines using quantitative PCR. (**B**) The VCAN gene was silenced using shRNA (shVCAN#1 and shVCAN#2) in BFTC909 cells, as determined by qPCR. (**C**) Western blot analysis was used to confirm VCAN silencing. (**D**,**E**) The migration of BFTC909 cells with shLuc, shVCAN#1 and shVCAN#2 was measured using an RTCA assay. (**F**,**G**) Cell proliferation was measured in BFTC909 cells with shLuc, shVCAN#1 and shVCAN#2 using an RTCA assay. Statistical analysis was performed by using one-way ANOVA with Tukey’s multiple comparisons test. ns, not significant; * *p* < 0.05; ** *p* < 0.01; *** *p* < 0.001.

To determine the most appropriate cells for our study, we examined the levels of VCAN expression in various urothelial cancer and UTUC cell lines. We collected five different cell lines, including SV-HUC-1, BFTC909, T24, J82 and UMUC-14 cells, and analyzed their VCAN RNA expression. The results showed that BFTC909, T24 and UMUC-14 cells had the highest VCAN RNA levels (Figure 5A). We then focused on using BFTC909 cells to investigate the role of the VCAN gene. We used shRNA to decrease VCAN gene expression and screened stable cells using puromycin. We were able to decrease VCAN expression by 96% at shVCAN#1 and 93% at shVCAN#2 (Figure 5B,C).

We used the Real-Time Cell Assay (RTCA) instrument to track and measure the migration of cancer cells in order to accurately assess the effect of suppressing VCAN. Results showed a significant decrease in cell migration when VCAN was suppressed in the BFTC909-shVCAN#1 and BFTC909-shVCAN#2 cells (Figure 5D). Further analysis of the slope results revealed that silencing VCAN significantly hindered cell migration at 18, 24, 48 and 72 h (Figure 5E). While the reduction in VCAN protein levels was not as significant with shVCAN#1 compared to shVCAN#2, this result was consistent with the subsequent cell migration results. Silencing of VCAN using shVCAN#2 had a more significant effect on cell migration, especially at 72 h. We investigated the impact of suppressing VCAN in cancer cells on cell proliferation, as cell proliferation affects instrument data. After 96 h, we found that suppressing VCAN had no effect on cancer cell proliferation (Figure 5F,G). This suggests that inhibiting VCAN in BFTC909 cells restricts cell migration without affecting cell proliferation, which is similar to the results in Figure 2C,D. VCAN is associated with lymphovascular invasiveness and tumor invasion stage in lymph nodes. 

### 2.6. Correlation Analysis of VCAN-Associated Gene Expression with Molecular Subtypes in UTUC Tumors

**Figure 6:**
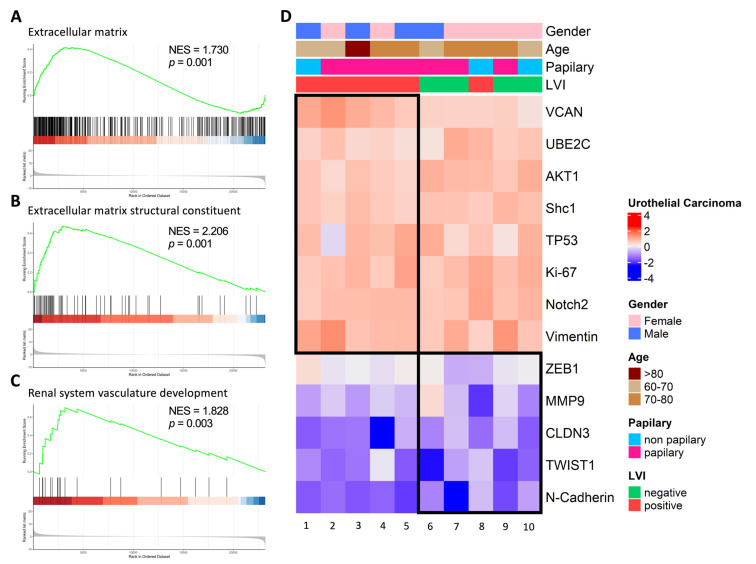
Analysis of the correlation between VCAN expression and molecular subtypes in UTUC. mRNA expression of molecular subtypes was analyzed based on the level of VCAN expression. (**A**–**C**) mRNA expression in ten patients with stage III UTUC tumors showed correlation with extracellular matrix organization, collagen-containing extracellular matrix and extracellular matrix structural constituents. (**D**) Gene clustering for migration biomarkers.

Our findings in Figure 1C indicated that upper urinary tract uroepithelial tumors were significantly associated with the organization of the extracellular matrix, collagen-containing extracellular matrix and structural components of the extracellular matrix, as determined by RNA sequencing analysis. We further analyzed the correlation between VCAN gene expression and the functions of the extracellular matrix in UTUC patients. We found that the RNA expression of patients with stage III tumors of UTUC was significantly associated with extracellular matrix organization, collagen-containing extracellular matrix and extracellular matrix structural constituents using GSEA analysis (Figure 6A–C). Additionally, we compared the expression levels of the VCAN gene between LVI(+) and LVI(−) subgroups and found that VCAN gene expression was significantly higher in the LVI(+) group (Figure 6D). We also observed differential expression of cancer-metastasis-inducing genes, such as ZEB1, MMP9, CLDN3, TWIST1 and N-cadherin, between LVI(+) and LVI(−) samples in some patients, indicating a correlation of VCAN with EMT genes in LVI(+) samples. Our findings suggest that the increased expression of the VCAN gene in the LVI(+) group may promote cell mobility by increasing the components of extracellular matrix organization, collagen-containing extracellular matrix and extracellular matrix structural constituents.

### 2.7. VCAN Enhanced Chemoresistance of Cancer Cells

**Figure 7:**
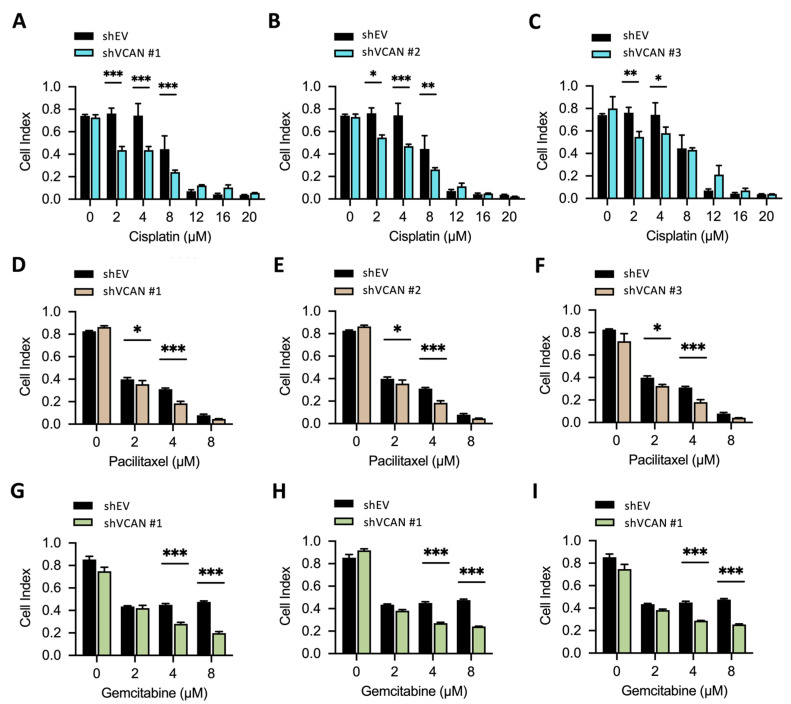
Silencing of VCAN increased drug sensitivity in UTUC. (**A**–**I**) BFTC909-shEV, shVCAN#1, shVCAN#2 and shVCAN#3 cells were treated with different concentrations of the drugs for 3 days, and the cell viability was then determined using the WST-1 assay. The data are presented as mean ± SD, and statistical analysis was performed using a two-way ANOVA with Sidak’s multiple comparisons test. * *p* < 0.05; ** *p* < 0.01; *** *p* < 0.001.

Our findings in Figure 5D indicated that reducing VCAN expression in UTUC cells can inhibit cell migration. To investigate the potential impact of this on treatment outcomes, we evaluated the effectiveness of various clinical drugs, including cisplatin, paclitaxel, gemcitabine and epirubicin, on UTUC cell growth. Cisplatin is a platinum-containing drug that hinders DNA replication and transcription, resulting in DNA damage and ultimately causing cell death. Paclitaxel and gemcitabine are nucleoside analog drugs with different mechanisms of action; paclitaxel stabilizes microtubules to halt cell division, while gemcitabine inhibits DNA synthesis to prevent cell replication and division. Epirubicin, an anthracycline drug, interferes with topoisomerase activity by intercalating DNA, producing free radicals and leading to DNA damage and cell death. We determined the half-maximal inhibitory concentration (IC50) of each drug on UTUC cells. BFTC909 cells were used for drug sensitivity tests. BFTC909-shEV, shVCAN#1, shVCAN#2 and shVCAN#3 cells were screened and treated with drugs in a dose-dependent manner. The shEV contains a scramble shRNA with mismatch sequences that do not target any specific gene. The cytotoxicity of cisplatin (2, 4 and 8 µM) was heightened by the suppression of VCAN (Figure 7A). The cytotoxicity of paclitaxel (2 and 4 µM) also increased upon VCAN silencing (Figure 7B). Similarly, the knockdown of VCAN raised the cytotoxicity of gemcitabine (4 and 8 µM) (Figure 7C). Our drug sensitivity test results indicate that VCAN inhibition in UTUC cells improves the effectiveness of clinical drugs such as cisplatin, paclitaxel and gemcitabine.

**Figure 8:**
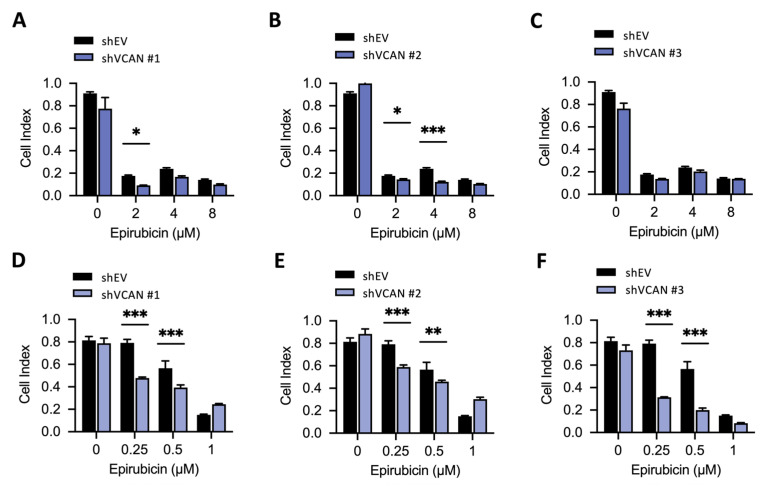
Silencing of VCAN enhanced the efficacy of low-dose epirubicin in UTUC. (**A**–**F**) BFTC909-shEV, shVCAN#1, shVCAN#2 and shVCAN#3 cells were treated with different concentrations of the drugs for 3 days, and the cell viability was then determined using the WST-1 assay. The data are presented as mean ± SD, and statistical analysis was performed using a two-way ANOVA with Sidak’s multiple comparisons test. * *p* < 0.05; ** *p* < 0.01; *** *p* < 0.001.

In order to examine the effects of VCAN inhibition on drug sensitivity in BFTC909 cells, we also treated the cells with a range of concentrations of epirubicin. Our results showed that treatment with 2 µM of epirubicin led to a significant amount of cell death (Figure 8A–C). To further investigate, we lowered the concentration of epirubicin and tested the effect of VCAN inhibition on drug toxicity. Our findings revealed that silencing VCAN using shRNA in BFTC909 cells enhanced the cytotoxicity of 0.25 and 0.5 µM epirubicin in three stable shVCAN cells (Figure 7G–I). These findings suggest that VCAN may be a potential target for UTUC treatment. 

## 3. Discussion

Our results demonstrated that the expression of VCAN was positively correlated with tumor stage, nodal metastasis status, histologic grade and histologic subtypes in the BLCA database. VCAN has previously been found to significantly increase in cases of lung inflammation [29,30,31]. VCAN regulates inflammation through the hyaluronic acid (HA) matrix [32,33]. Monocytes/macrophages and stromal fibroblasts are capable of secreting VCAN, which is involved in the formation of the HA matrix. Additionally, VCAN is considered as a cancer-causing factor in various types of cancer, including pancreatic cancer, myeloma, ovarian cancer, hepatocellular carcinoma, colon carcinoma, glioma and bladder cancer [22,34,35,36,37,38,39,40]. The role of VCAN in UTUC has not been reported previously, but our results suggest that VCAN expression has a significant relationship with the pathological features and patient survival in UTUC.

In breast cancer, some researchers have discovered that platelet-derived growth factor (PDGF) stimulates arterial smooth muscle cells to express VCAN, leading to an increase in the expression of ECM components in surrounding cells, thereby promoting the growth and metastatic ability of cancer cells [41,42,43]. Another study in breast cancer indicates that VCAN has an anti-apoptotic effect, as it strengthens the binding between cells and the extracellular matrix (ECM) and promotes integrin beta1 to enhance cell adhesion, thereby reducing cell death caused by oxidative stress [44]. LaPierre et al. have reported that VCAN plays a role in apoptotic resistance in some tumors, while it acts as a sensitization factor in others. Currently, it has been found that VCAN may regulate the activity of the tumor suppressor p53 and Mdm2, thus affecting sensitivity to apoptosis [45]. These results show that the pathological significance and function of VCAN depends on the type of cancer cells and has different cell survival characteristics due to differences in gene functions involved in the response.

In summary, VCAN, which regulates inflammation through the hyaluronic acid matrix, has been shown to contribute to the development and metastasis of different types of cancer, such as breast and bladder cancers. The expression of VCAN stimulates arterial smooth muscle cells, promoting the growth and metastasis of breast cancer cells, and has an anti-apoptotic effect, reducing cell death caused by oxidative stress. However, VCAN has different cell survival characteristics due to differences in gene functions involved in the response, acting as a sensitization factor in some tumors and playing a role in apoptotic resistance in others. Therefore, the pathological significance and signaling processes mediated by VCAN differ among different cancer cells. Our results revealed that VCAN promoted ECM structural composition and molecular mechanisms such as collagen expression in UTUC and was associated with invasion and metastatic signaling molecules (Figure 1 and Figure 6), indicating that the mechanisms of action of the same gene can also vary across cancer types. VCAN is a large protein (chondroitin sulfate proteoglycan) with a molecular weight exceeding 1000 kD [46]. These findings highlight VCAN’s multiple regulatory mechanisms in tumors and suggest that it may promote tumor growth or migration through different protein interactions on various regions of the protein.

To our knowledge, this is the first study on the expression of VCAN and its impact on prognosis and lymphatic metastasis in UTUC patients. Methylation detection is commonly used in tumors to measure the levels of oncogenic and anti-oncogenic genes, and it is highly correlated with gene expression [47,48]. We found that UTUC patients with positive lymphatic metastasis often have a poor prognosis, and the degree of methylation in the VCAN intron 2 measured in chromosome methylation analysis can be a predictive factor for UTUC patients with positive lymphatic metastasis (Figure 3).

Previous studies have reported a correlation between VCAN expression and the immune microenvironment in cervical cancer patients. In high-expression cervical cancers, there is a significant decrease in CD8^+^ T cells around epithelial cells [49]. There is some evidence indicating a potential association between VCAN and distant metastases. In a study involving 4T1 mouse mammary carcinoma, it was found that the expression level of VCAN is related to the number of tumor-associated macrophages (TAM) in the tumor microenvironment and can promote lung lymph node metastasis [50]. A study using a mouse mammary tumor virus-polyoma middle T-antigen (MMTV-PyMT)-induced cancer model also demonstrated that high expression of VCAN promotes cancer cell metastasis to the lung in a TGF-β-dependent manner [51]. However, there are few reports on the exploration of VCAN in cancer patients and its interaction with the immune microenvironment. Our results indicate that VCAN expression is significantly associated with positive lymphatic metastasis in multiple analyses, including pathological characteristics. Further studies are needed to determine the immune microenvironment of positive lymphatic metastasis patients and whether VCAN affects the expression of immune cells.

Previous research reports have indicated that VCAN promotes the generation of new blood vessels in glioblastoma [52]. There have also been reports finding that tumors with high expression of VCAN contain more fiber-connecting proteins and VEGF, which stimulates endothelial cell adhesion and growth and migration [53]. Additionally, in vitro and in vivo studies of breast cancer have also shown that VCAN interacts with the microenvironment’s matrix, fibronectin and VEGF to promote angiogenesis [54]. However, there have been limited reports on the role of VCAN in cancer lymphatic metastasis. Our results provide evidence of VCAN’s role in tumor lymphatic metastasis and a method to measure its expression using chromatin methylation at specific loci.

According to the findings of Tomiyama et al., the mutation rates of TP53 and ERBB2 were positively correlated with high-grade UTUC patients [55]. Our heatmap analysis revealed a correlation between the expression of VCAN and the cell migration genes MMP9, TWIST1 and N-cadherin in UTUC patients (Figure 6). According to other studies on UTUC, the expression of MMP9 in malignant cells is associated with the tumor suppressor gene WWC1. In addition, in UTUC, the use of MiR-26a-5p to inhibit WNT5A/β-catenin signaling affects the EMT process, significantly suppressing the cells’ invasion ability by downregulating the expression of NF-κB and MMP-9 [56].

One of the most interesting results of this study is that the absence of VCAN can enhance the efficacy of drugs such as cisplatin, gemcitabine and epirubicin (Figure 7 and Figure 8), indicating the potential of inhibiting VCAN to amplify the effects of these drugs. Further research is needed to identify the genes affected by inhibiting VCAN that synergistically promote the action of clinical drugs.

In conclusion, VCAN expression was positively correlated with the tumor stage, nodal metastasis status, histologic grade, and histologic subtypes in UTUC patients. Additionally, its expression and chromosomal methylation level were useful markers for predicting positive lymphatic metastasis. The molecular mechanism of VCAN’s anti-cancer function suggests that it can promote tumor cell migration and EMT-related molecular expression. The potential for inhibiting VCAN to enhance the effects of drugs such as cisplatin, gemcitabine and epirubicin shows that VCAN could potentially serve as a biomarker to identify patients who are at a higher risk of developing cancer or who may respond better to drug therapies. Additionally, it may be used as a target for the development of new drugs or therapies that can specifically inhibit the expression of the VCAN.

## 4. Materials and Methods

### 4.1. Tissue Samples

UTUC samples were gathered from patients at the Kaohsiung Chang Gung Memorial Hospital. The research project was approved by the Chang Gung Medical Foundation Institutional Review Board (IRB number: 201504731B0 and 202100375B0).

### 4.2. Immunohistochemistry

The human UTUC tissue samples used in the study were obtained from the tissue bank at the Chang Gung Memorial Hospital in Kaohsiung, Taiwan. The tissues were fixed in formalin, embedded in paraffin and cut into sections. An automated Bond-Max instrument (Leica Microsystems, Wetzlar, Germany) was used for tissue immunostaining following the instructions provided by the manufacturer. The following steps were performed with the following settings: (1) dewaxing: glass slides were rinsed with dewaxing solution at 72 °C. (2) Antigen retrieval: tissue slides were immersed in antigen retrieval buffer at 100 °C for 10 min. (3) Peroxide block: glass slides were immersed in hydrogen peroxide solution and left to react at room temperature for 10 min. (4) Primary antibody reaction: VCAN (1:100) was applied and maintained at room temperature for 60 min. (5) Post primary reagent reaction: this step was performed at room temperature for 10 min. (6) DAB staining: 3,3′-diaminobenzidine tetrahydrochloride (DAB) was used for 3 min at room temperature. (7) Counterstain: hematoxylin was applied for 1 min. After the tissue slides were mounted, they were scanned using the Vectra Polaris Automated Quantitative Pathology Imaging System (PerkinElmer, Boston, MA, USA), and the staining level was quantified using the inForm Advanced Image Analysis Software (version 2.3, PerkinElmer). The tumor morphology and VCAN levels on the slides were independently assessed by a urological pathologist (Dr. Min-Tse Sung) and a urological oncologist (Dr. Hao-Lun Luo) at the Chang Gung Memorial Hospital.

### 4.3. Cell Lines, shRNA, RNA Isolation and Quantitative Real-Time PCR 

The SVHUC-1 (CRL-9520, ATCC), J82 (HTB-1, ATCC), BFTC909 (60069, BCRC) and T24 (60062, BCRC) cell lines were obtained from the American Type Culture Collection (ATCC) and the Bioresource Collection and Research Center (BCRC). RNA knockdown was performed using shRNA virus from the RNAi Core at Academic Sinica in Taiwan. The shRNA targeting sequences for VCAN were shVCAN#1: GCCACAGTTATTCCAGAGATT and shVCAN#2: ATGGATGTGTTCAACCTTAAT. ShEV was a synthetic shRNA that contained a scrambled sequence with a few mismatches, generated by inserting a 53-mer oligonucleotide (agttcagttacgatatcatgtctcgagacattcgcgagtaactgaacttttttg). The RNA present in the UTUC clinical tissues and cells were purified using the QIAGEN RNA purification kit. The RNA was then purified and subjected to reverse transcription using 5 micrograms of RNA per sample with the PrimeScript™ RT Reagent Kit (TaKaRa, Shiga, Japan). The resulting cDNA was ready for quantitative real-time PCR. Quantitative real-time PCR was performed using SYBR Green PCR master mix and an ABI 7500 sequence detection system (Life Technologies, Carlsbad, CA, USA). The real-time PCR primers for VCAN were: forward: 5′-TTGGACCTCAGGCGCTTTCTAC-3′; reverse: 5′-GGATGACCAATTACACTCAAATCAC-3′. The GAPDH primers were: forward: 5′- TGAAGGTCGGAGTCAACGGATT-3′; reverse: 5′-CCTGGAAGATGGTGATGGGATT-3′.

### 4.4. Western Blotting Analysis

The cells were lysed in a modified RIPA buffer that contained a protease inhibitor mixture (Roche, Basel, Switzerland). The protein concentration in the supernatant was measured relative to standard BSA concentrations using a BCA protein assay kit (Pierce, Waltham, MA, USA). Thirty micrograms of cell lysate protein were loaded per lane of a 10% SDS-PAGE gel. The protein was then separated and transferred onto an Immobilon-p Transfer Membrane (Millipore, Darmstadt, Germany). The membranes were probed with specific antibodies against VCAN (12C5, 1:1000 dilution, DSHB, Iowa, IA, USA) and GAPDH (MAB374, 1:1000 dilution, Millipore) overnight and for 2 h, respectively. After a 1 h reaction with a horseradish-peroxidase-conjugated secondary antibody (1:2000 dilution, Cell Signaling Technology, Beverly, MA, USA), each membrane was scanned using a UVP ChemStudio PLUS instrument (UVP Inc., Upland, CA, USA) and analyzed with ImageJ software (version 1.8).

### 4.5. Whole Transcriptome RNAseq

The gene expression in all tumor tissues was analyzed by conducting whole transcriptome sequencing on Illumina HiSeq 2500 sequencers as per the manufacturer’s guidelines. The Cufflinks pipeline was utilized for the gene expression analysis of each sample based on the aligned RNAseq reads. The preparation of the reads was conducted using the Tophat (v2.1.1)’s “prep_reads” software tool, considering the previously calculated fragment size distributions and Ensemble gene annotations (GRCh38.p13). The alignment was carried out with the help of Tophat (v2.1.1) and Bowtie2, taking into account the estimated fragment size distributions and Ensemble gene annotations. The expression of each Ensemble gene was estimated by Cufflinks (v2.2.1) with the following parameters: “library-type fr-first strand” and “multi-read-correct.”

### 4.6. Methylation-Specific PCR

The DNA methylation analysis was carried out based on previous literature (36). In brief, chromosome DNA was obtained from normal and tumor tissue samples (n = 10 each) using the QIAamp DNA Mini Kit (Qiagen, Valencia, CA, USA). The primers for methylation-specific PCR were designed using the MethPrimer (https://www.urogene.org/methprimer/, accessed on 21 March 2022). The methylation primers for VCAN were: forward: 5′-GAGAGGTTAAGTAGTTTGTTCGAAC-3′; reverse: 5′-TAAAATACAATAATACAATCCCGAC-3′. Next, 500 ng of DNA was converted through the EZ DNA Methylation Kits of Bisulfite Conversion System (Zymo Research, Irvine, CA, USA). The bisulfite conversion was conducted in the dark at 50 °C for 16 h, and then the transformed DNA was desulfurized. Then 20 μL of elution buffer was added to elute the converted DNA. CpG methylation levels were detected using PCR amplification with the HotStarTaq^®^ MasterMix kit (Qiagen, Cat. No. 203443). The amplification conditions were 95 °C for 1 min, 90 °C for 30 s, 55–60 °C for 30 s, 72 °C for 30 s and 40 cycles. The relative methylation levels of each CpG site were analyzed using the Image J software (version 8).

### 4.7. RTCA Cell Proliferation Assay

The cell proliferation assay was performed based on the experimental conditions of Roshan Moniri et al. [57]. The number of cells for BFTC909, T24 and J82 were 5000 cells per well. Each experiment was conducted three times and monitored using the RTCA instrument for 96 h. The following parameters were set: the first step was a blank test, which was carried out every one minute for a total of 11 tests. In the second step, the cells were tested every 15 min for a total of 400 tests. For the blank test, the E plate was used, and 50 μL of 12% DMEM was added to each well. The experiment commenced by adding 150 μL to each well containing 5000 cells. After 96 h, the experimental results were quantified and subjected to statistical analysis.

### 4.8. RTCA Cell Migration Assay

The cell migration assay was carried out based on the experimental conditions specified by Roshan Moniri et al. [57]. Each experiment was conducted three times and monitored using the RTCA instrument for 96 h. The blank test was performed using a CIM-plate, where 160 μL of DMEM with 12% FBS was added to each well of the lower chamber, and 50 μL of serum-free DMEM was added to each well of the upper chamber. The CIM-plate was incubated at 37 °C in an incubator for 1 h. Then, 100 μL of serum-free DMEM was added to each well containing 20,000 cells. After 96 h of migration, the experimental results were quantified and subjected to statistical analysis.

### 4.9. Drug Sensitivity Assay

Cells were seeded in triplicate in 96-well plates (20,000 cells/well) and incubated for 24 h. Drugs were added at concentrations ranging from 0.25 µM to 8 µM with DMSO as a control. Both the control group and the experimental group cells were cultured in puromycin, and puromycin treatment was terminated 2–3 days before drug treatment. Cell viability was evaluated using the WST-1 Kit (Thermo Fisher Scientific, Waltham, MA, USA) after 3 days of drug treatment and analyzed statistically using GraphPad Prism 8 software (GraphPad, San Diego, CA, USA).

### 4.10. Statistical Analysis

All in vitro experiments were performed at least three times, and the results were presented as the mean ± SD unless stated otherwise. A *p*-value less than 0.05 was considered to be statistically significant. The data obtained from the methylation-specific PCR assay, proliferation assay, real-time PCR analysis and drug sensitivity assay were expressed as mean ± SD. The difference between different experimental groups was analyzed using a two-tailed Student’s *t*-test. The statistical analysis was conducted using GraphPad Prism 8 software (GraphPad, San Diego, CA, USA).

## Data Availability

The data presented in this study are available on request from the corresponding author. The data are not publicly available due to privacy.

## Supplementary Materials

The supporting information can be downloaded at: https://www.mdpi.com/article/10.3390/ijms24087486/s1.
