# Supplementary material for: VCAN Hypomethylation and Expression as Predictive Biomarkers of Drug Sensitivity in Upper Urinary Tract Urothelial Carcinoma
Source: Int J Mol Sci. 2023 Apr 19;24(8):7486. doi: 10.3390/ijms24087486 (PMC10139123; doi:10.3390/ijms24087486)
Supplement: Supplementary file 1 [file ijms-24-07486-s001.zip › ijms-2272944-supplementary.pdf]

Supplementary Figure S1

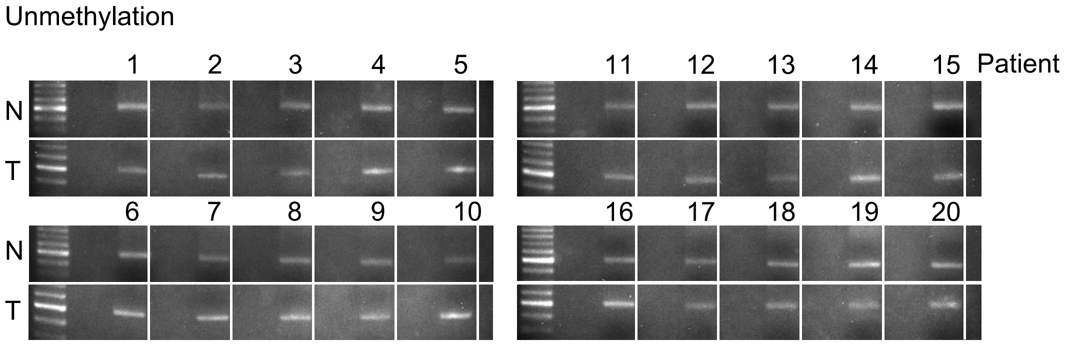

Supplementary Figure S1. DNA agarose gel analysis was performed to quantify the unmethylation in site 4 of VCAN gene from N/T pairs of UTUC.
